# Risk factors for death among children aged 5–14 years hospitalised with pneumonia: a retrospective cohort study in Kenya

**DOI:** 10.1136/bmjgh-2019-001715

**Published:** 2019-09-03

**Authors:** Liana Macpherson, Morris Ogero, Samuel Akech, Jalemba Aluvaala, David Gathara, Grace Irimu, Mike English, Ambrose Agweyu

**Affiliations:** 1 Health Services Unit, KEMRI-Wellcome Trust Research Programme, Nairobi, Kenya; 2 Department of Paediatrics and Child Health, University of Nairobi, Nairobi, Kenya; 3 University of Nairobi College of Health Sciences, Nairobi, Kenya; 4 Nuffield Department of Clinical Medicine, Oxford University, Oxford, UK

**Keywords:** infectious diseases, epidemiology, pneumonia

## Abstract

**Introduction:**

There were almost 1 million deaths in children aged between 5 and 14 years in 2017, and pneumonia accounted for 11%. However, there are no validated guidelines for pneumonia management in older children and data to support their development are limited. We sought to understand risk factors for mortality among children aged 5–14 years hospitalised with pneumonia in district-level health facilities in Kenya.

**Methods:**

We did a retrospective cohort study using data collected from an established clinical information network of 13 hospitals. We reviewed records for children aged 5–14 years admitted with pneumonia between 1 March 2014 and 28 February 2018. Individual clinical signs were examined for association with inpatient mortality using logistic regression. We used existing WHO criteria (intended for under 5s) to define levels of severity and examined their performance in identifying those at increased risk of death.

**Results:**

1832 children were diagnosed with pneumonia and 145 (7.9%) died. Severe pallor was strongly associated with mortality (adjusted OR (aOR) 8.06, 95% CI 4.72 to 13.75) as were reduced consciousness, mild/moderate pallor, central cyanosis and older age (>9 years) (aOR >2). Comorbidities HIV and severe acute malnutrition were also associated with death (aOR 2.31, 95% CI 1.39 to 3.84 and aOR 1.89, 95% CI 1.12 to 3.21, respectively). The presence of clinical characteristics used by WHO to define severe pneumonia was associated with death in univariate analysis (OR 2.69). However, this combination of clinical characteristics was poor in discriminating those at risk of death (sensitivity: 0.56, specificity: 0.68, and area under the curve: 0.62).

**Conclusion:**

Children >5 years have high inpatient pneumonia mortality. These findings also suggest that the WHO criteria for classification of severity for children under 5 years do not appear to be a valid tool for risk assessment in this older age group, indicating the urgent need for evidence-based clinical guidelines for this neglected population.

**Key questions**

**What is already known?**

- The evidence on risk factors for death in children >5 years with community-acquired pneumonia is extremely limited, and definitions for what constitutes severe disease are not standardised.
- In fact, there are no studies from low-income or middle-income countries that report risk factors for inpatient pneumonia mortality specifically in children >5 years.
- As such, data to inform age-specific case management in low-income and middle-income settings do not exist.

**What are the new findings?**

- Pneumonia mortality in this cohort is high (7.9%).
- The risk factors for pneumonia mortality in children >5 years are broadly similar to those identified for children under 5 years.
- These include: the presence of severe pallor, reduced level of consciousness, mild/moderate pallor, central cyanosis, older age (>9 years), respiratory rate >30/min, inability to eat or drink, HIV and severe malnutrition.

**What do the new findings imply?**

- While these data do not constitute formal evidence that using these patient features to indicate high risk of mortality would save lives, they do make it clear that we need to better understand the characteristics of children presenting with possible pneumonia at all levels of the health system and that there is a need for research to explore the underlying factors that account for this.

## Introduction

In 2017, there were still an estimated 6.3 million child deaths globally, of which, almost 1 million occurred in children aged 5–14 years.^1^ According to a recent review of mortality trends in this older age group, 98% of deaths occurred in low-income or middle-income countries (LMICs) and 55% occurred in sub-Saharan Africa—an increase from 36% in 1990.^1 2^ In sub-Saharan Africa, the probability of a child aged 5 years dying between the ages of 5 and 15 years was reported at 18.4 deaths per 1000 children, which is 17 times higher than in high-income countries.^2^ Historically, global initiatives to reduce child mortality have focused on children under 5 years. This was reflected in the United Nation’s Millennium Development Goals and again in their Sustainable Development Goals (2015). In contrast, morbidity and mortality among older children and adolescents have received little attention.

The limited data available suggest that lower respiratory tract infections account for a significant proportion of deaths for children aged 5–14 years.^3 4^ However, the WHO pneumonia case-management guidelines are specific to children under 5 years.^5^ The WHO’s Integrated Management of Adolescent and Adult Illness (IMAI) initiative does provide guidance and training material for older patients,^6^ but these are based on limited evidence, and group all those aged over 5 years into just one category. Children over 5 years may require specific management, for example, some data suggest that they are more likely to present with atypical infection.^7^ However, in low-resource settings, age-specific evidence to inform clinical risk assessment and treatment policies is lacking, and is much needed to improve quality of care.

In this analysis, we describe the clinical characteristics and comorbidities of children aged 5–14 years admitted to district-level hospitals in Kenya with pneumonia, and assess risk factors for death. We highlight clinical features that may improve risk assessment and examine the performance of the existing WHO criteria (intended for under 5s) for differentiating children at risk of death to inform potential future studies on appropriate guidelines in this age group. Finally, we explore current prescription patterns in older children for whom no national guidance is available in Kenya.

## Methods

### Study design, setting and population

We did a retrospective cohort study using routine clinical data from an established Clinical Information Network (CIN) in Kenya and examined the association between individual clinical characteristics and inpatient mortality using logistic regression. We reviewed inpatient records for all children age 5–14 years admitted to hospital over a 4-year period—1 March 2014 to 28 February 2018—with a clinician diagnosis of pneumonia at the point of discharge or death. Children 15 years and over were excluded, as hospital policies would dictate that these patients are admitted under adult care.

The CIN was established in September 2013 to foster better generation and use of clinical information and improve quality of care.^8 9^ The network consists of 13 purposely selected public county hospitals, situated in regions of high and low malaria transmission, which are representative of district-level health facilities in Kenya. They have limited diagnostic capacity, including poor access to pulse oximetry and microbiological investigation, and diagnoses are generally made by junior clinicians who review all children requiring admission and prescribe their initial treatment. As a component of the network intervention, quality of inpatient care is periodically audited against national protocols,^10^ which are adapted from the WHO guidelines.^5 11^ This is fed back to the hospitals for quality improvement,^12^ although given the lack of clear guidelines for children over 5 years, this feedback is limited to under 5 conditions. Routine data collected includes: biodata, clinical information, history and examination findings, admission and discharge diagnoses, investigations, treatments prescribed and outcomes. All clinical variables are collected at admission, discharge diagnosis and outcome are collected at the point of discharge (including death) and investigations (malaria and HIV) are captured at various points during the inpatient stay.

### Patient and public involvement

Not applicable as this study was a secondary analysis of routinely collected data.

### Pneumonia severity

We examined individual clinical risk factors for association with mortality (detailed in the ‘Statistical analysis’ section). To explore whether the existing WHO criteria were useful in discriminating risk in this older cohort, we used the available information on clinical characteristics to define two levels of severity based on the 2013 WHO pneumonia guidelines for use in children under 5 years.^5^ The details of our approach are provided in table 1. We were unable to include oxygen saturations (<90%) in the study definition for severe disease, as pulse oximetry was not consistently available in the CIN hospitals. The WHO guidelines for children under 5 years and the IMAI guidelines are also presented in table 1 for comparison.

**Table 1:** WHO under 5, IMAI and operational study definitions of pneumonia

	Clinical signs *Adapted from WHO Pocket Book of Hospital Care for Children for children under 5 years* 5	Clinical signs Adapted from the IMAI District Clinician manual for adolescents and adults* 6	Clinical signs *Operational study definitions*
***Severe pneumonia*** *Recommended treatment:* *intravenous* *p* *enicillin with* *g* *entamicin* *†*	**History of cough or difficulty breathing and any one of:** Oxygen saturations <90%, or central cyanosis Severe respiratory distress General danger sign (inability to breast feed or drink, lethargy or reduced level of consciousness, convulsions)	**Any one of:** Appears obstructed Central cyanosis Severe respiratory distress **AND any one of:** Oxygen saturations <90% Respiratory rate >30/min Fever or suspected infection Signs of severe respiratory distress	**Pneumonia diagnosis and any one of:** Central cyanosis Severe respiratory distress (grunting, stridor) General danger sign (inability to feed or drink, reduced level of consciousness)
**Non-severe pneumonia** *Recommended treatment: intravenous penicillin or oral amoxicillin†*	**History of cough or difficulty breathing and** Fast breathing (for age) or Chest indrawing Without any of the criteria for severe pneumonia	Signs and symptoms of pneumonia without meeting the criteria for severe pneumonia	Pneumonia diagnosis without meeting the criteria for severe pneumonia

*The assessment for severe pneumonia comes after an initial ‘quick check’ using the airway, breathing, circulation approach to identify patients presenting with emergency signs and is not intended for those with features of shock.

†These are the recommendations as per the WHO Pocket Book of Hospital Care for Children.^5^

IMAI, Integrated Management of Adolescent and Adult Illness.

### Comorbidities

We identified children with comorbidities using a combination of documented discharge diagnoses, clinical characteristics at presentation and laboratory data (table 2). We examined HIV, sickle cell disease, chronic neurological disorder, asthma, cardiac disease, renal disease, severe malnutrition, malaria, sepsis, acute neurological disease and diarrhoea with dehydration for association with death. To identify children with severe malnutrition we used the clinician diagnosis, or the presence of any one of: oedema (not attributable to renal disease); mid-upper arm circumference (MUAC) ≤115 mm; weight-for-height z (WHZ) score ≤−3 SD. We were unable to use data for MUAC and height alone to identify children with malnutrition as these measures were poorly recorded (missing in 60% and 76% of cases, respectively).

**Table 2:** Study classification of comorbidities

Comorbidity	Study classification
Chronic neurological disorder, asthma, cardiac disease, renal disease, sepsis	Documented clinician diagnosis.
Sickle cell disease, malaria	Documented clinician diagnosis or any positive laboratory result.
HIV	Documented clinician diagnosis or any positive HIV test result (serology or PCR).
Severe malnutrition	Documented severe malnutrition clinician diagnosis or oedema, MUAC <115 mm, WHZ ≤−3 SD.
Acute neurological disorder	Documented meningitis clinician diagnosis of meningitis or history of convulsions or altered level of consciousness based on the AVPU scale.
Diarrhoea with dehydration	Documented clinician dehydration diagnosis and history of diarrhoea.

AVPU, alert, verbal, pain, unresponsive; MUAC, mid-upper arm circumference; WHZ, weight-for-height z score.

### Treatment

In the absence of treatment guidelines for children aged 5–14 years, we used available information on admission prescriptions to describe antibiotic use for our cohort and explore whether children were treated as per the WHO pneumonia guidelines for children under 5 years.^5^ For the purpose of this latter analysis only, children who were re-admitted (within 30 days of the presenting illness episode) or were referred from another hospital, and those with HIV or severe malnutrition were excluded, as WHO recommendations for first-line antibiotic therapy are not directly applicable to these groups. WHO under 5 guidelines recommend that children presenting with signs of severe pneumonia should receive intravenous penicillin and gentamicin, and those with non-severe pneumonia should receive oral amoxicillin or, in pre-2013 guidelines, intravenous penicillin monotherapy (table 1). In our setting, amikacin is often used when gentamicin is unavailable so the two were combined under the heading aminoglycosides. We were unable to account for the effect of antibiotic treatment on outcome because we only had information on *prescriptions* on *admission* which does not reflect what was actually administered, nor any changes to treatment made during admission.

### Data management

Within the CIN, clinician diagnoses are documented according to the 10th Revision of International Statistical Classification of Diseases and Related Health Problems system. For pragmatic reasons, the research team reclassified these diagnoses to fit one of 38 predefined categories. Clinical information is captured through standardised, Ministry of Health approved, Paediatric Admission Record forms. These are used by clinicians and form part of the medical records, they prompt the clinician for information with a series of checkboxes and free text options. At the end of each admission episode (discharge from hospital or death), data are abstracted directly from the patient’s hospital records—medical notes, nursing charts, treatment charts and laboratory reports—and are recorded directly into the primary data collection tool (developed in Research Electronic Data Capture).^13^ Data are entered retrospectively at each hospital by a trained clerk with remote support from a central data team. This process has automated point of entry error checks and regular external data quality assurance. Descriptive analyses on the magnitude and nature of the data errors captured from this network indicate that errors are uncommon.^14^ In some hospitals with high work load, a minimum set of data is collected for a random sample of patients. These data only include variables that are required for standard health system reports and have limited clinical information, therefore, these patients were not included in this analysis. A more detailed description of the methodology for collection and cleaning of hospital data within the CIN is described elsewhere.^15^

Our findings were reported in keeping with the Strengthening the Reporting of Observational Studies in Epidemiology statement guidelines,^16^ and Ethical approval for the use of de-identified routine data was provided by the parent study of the CIN from the Kenya Medical Research Institute (KEMRI) Scientific and Ethics Committee (SERU), approval numbers SSC 1746 and 2771). The Ministry of Health and participating hospitals also gave their permission.

### Statistical analysis

Continuous variables were reported as mean (SD) or median (IQR) as appropriate, categorical data were summarised as proportions. We used χ^2^ test to determine associations between categorical variables, except where the sample size was ≤5 where Fisher’s exact test was applied. Univariate associations with mortality were calculated for demographic and clinical characteristics, and comorbidities. Factors found to be associated with mortality in univariate analysis (p<0.1) and with <20% missing data were then considered for inclusion in the multivariate models. Age and sex were included a priori and hospital identity was included as a random effect. In *model 1*, clinical characteristics were examined for association with death. In *model 2*, information on HIV, malaria and severe malnutrition was incorporated. These comorbidities were agreed a priori and were selected due to their clinically important impact on outcome and because they can be diagnosed early during the admission and could feasibly form part of a clinical risk assessment strategy. The models were built sequentially to mirror information available to the clinician from the time the child is first assessed to the availability of rapid diagnostic test results and assessment of nutritional status. At each stage, variables for inclusion in the multivariate models were reviewed and excluded if they expressed collinearity with another variable. The following interactions were also examined: malaria and pallor; age and HIV; age and pallor.

We performed a separate analysis to explore the effect of nutritional status defined by weight-for-age z scores (WAZ) on clinical outcome (online supplementary material *model 3*). WAZ scores are often used to describe nutritional status in studies of children under 5 years,^17 18^ and these variables (weight and age) are well recorded in our data. We calculated WAZ scores using WHO child growth standards,^19^ excluding children over 10 years where there are no reference standards (WAZ scores are not reliable in the context of pubertal growth spurts).^19^ We also performed an adjusted subgroup analysis of children from non-malaria endemic regions to explore the effect of pallor on outcome in a population where malaria is unlikely to account for anaemia (online supplementary material *model 4*).

10.1136/bmjgh-2019-001715.supp1 Supplementary data

To handle missing data, we performed multiple imputation with chained equations for variables with <20% missingness. We generated 30 imputed datasets, each with 100 iterations, under the assumption that data were missing at random. Finally, we calculated the sensitivity, specificity, positive and negative likelihood ratios, and area under the receiver operator curve in predicting death for severe pneumonia and for the clinical characteristics that were associated with mortality in adjusted analyses (online supplementary table 1). All statistical analyses were done using R statistical computing software V.3.4.4.^20^

## Results

**Figure 1:**
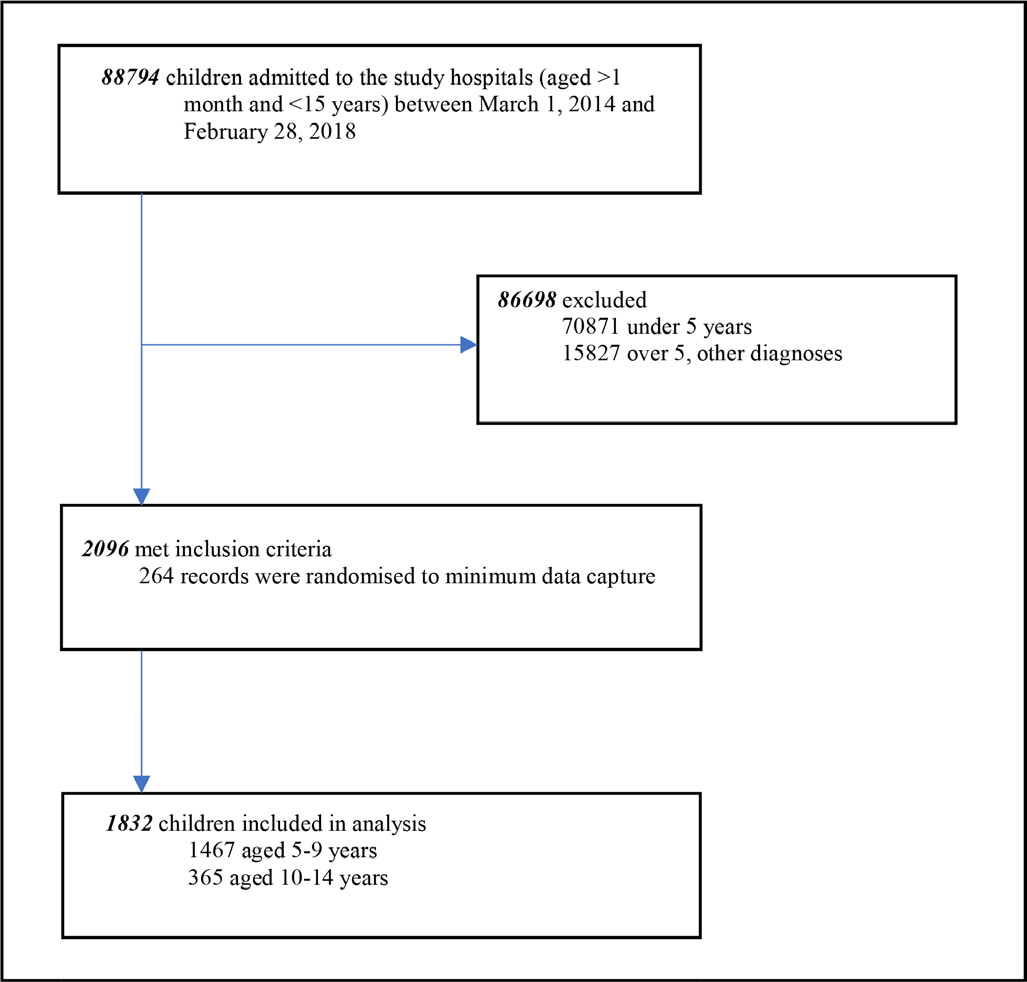
Age distribution (years) of children admitted to the study hospitals with pneumonia.

**Figure 2:**
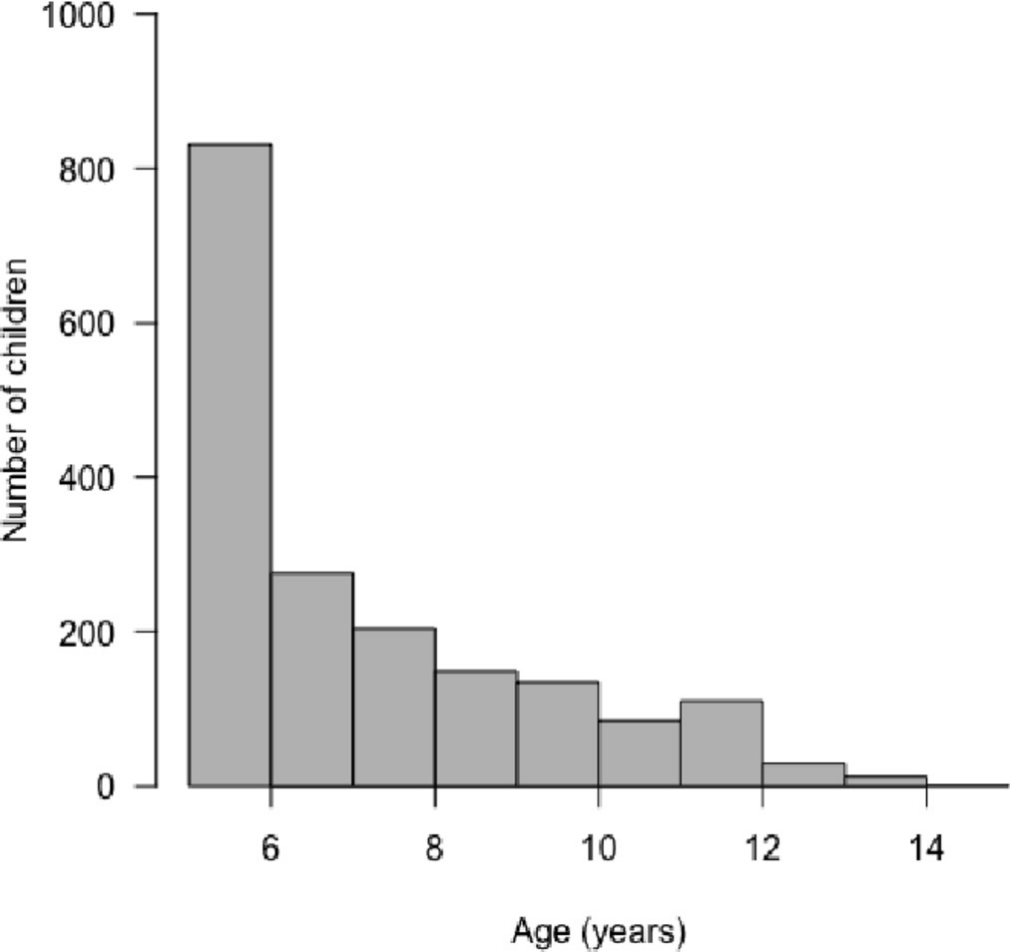
Age distribution (years) of children admitted to the study hospitals with pneumonia.

There were 88 794 children aged >1 month to <15 years admitted to the study hospitals between 1 March 2014 and 28 February 2018, 17 923 (20.2%) were over 5 years. Of those over 5 years, 2096 (11.7%) had a discharge diagnosis of pneumonia. Two hundred sixty-four patient records were randomised to have minimum data captured. Therefore, analyses included 1832 children (figure 1). The median age of the study population was 84 months (IQR 66–108) and the majority (80%) of children in the study were aged 5–9 years (figure 2). Hospital location was available for all patients and 849 (46.3%) were admitted to hospitals in high malaria transmission regions of Kenya. Clinical characteristics are shown in table 3.

**Table 3:** Characteristics and comorbidities of children admitted to study hospitals with pneumonia

Characteristic	Age 5–9 years n=1467*	Age 10–14 years n=365*	Total n=1832*
Demographic information			
Male	836/1461 (57.22%)	185/365 (50.68%)	1021/1826 (55.91%)
Hospitals in high malaria transmission region	641/1467 (43.69%)	208/365 (56.99%)	849/1832 (46.34%)
Referred to hospital	239/1214 (19.69%)	85/297 (28.62%)	324/1511 (21.44%)
Readmitted to hospital	232/1232 (18.83%)	47/296 (15.88%)	279/1528 (18.26%)
Clinical characteristic			
Temperature ≥38°C	559/1321 (42.32%)	111/318 (34.91%)	670/1639 (40.88%)
Respiratory rate >30/min	937/1216 (77.06%)	191/291 (65.64%)	1128/1507 (74.85%)
Central cyanosis	24/1418 (1.69%)	1/353 (0.28%)	25/1771 (1.41%)
Grunting	232/1394 (16.64%)	39/348 (11.21%)	271/1742 (15.56%)
Acidotic breathing	58/1382 (4.2%)	12/350 (3.43%)	70/1732 (4.04%)
Reduced consciousness	108/1400 (7.71%)	23/348 (6.61%)	131/1748 (7.49%)
Cannot eat or drink	235/1347 (17.45%)	62/332 (18.67%)	297/1679 (17.69%)
Stridor	46/1356 (3.39%)	12/342 (3.51%)	58/1698 (3.42%)
Difficulty breathing	743/1416 (52.47%)	159/352 (45.17%)	902/1768 (51.02%)
Wheeze	189/1396 (13.54%)	34/350 (9.71%)	223/1746 (12.77%)
Crackles	504/1387 (36.34%)	109/351 (31.05%)	613/1738 (35.27%)
Severe pallor	89/1411 (6.31%)	26/349 (7.45%)	115/1760 (6.53%)
Mild/moderate pallor	158/1411 (11.2%)	67/349 (19.2%)	225/1760 (12.78%)
No pallor	1164/1411 (82.49%)	256/349 (73.35%)	1420/1760 (80.68%)
Chest wall indrawing	609/1400 (43.5%)	102/349 (29.23%)	711/1749 (40.65%)
Convulsions	212/1406 (15.08%)	42/347 (12.1%)	254/1753 (14.49%)
Oxygen saturation <90%	151/661 (22.84%)	29/151 (19.21%)	180/812 (22.17%)
Mid-upper arm circumference <115 mm	41/620 (6.61%)	6/113 (5.31%)	47/733 (6.41%)
Severe pneumonia	492/1443 (34.1%)	96/356 (26.97%)	588/1799 (32.68%)
Comorbidity			
HIV positive	122/1467 (8.32%)	60/365 (16.44%)	182/1832 (9.93%)
Malaria diagnosed	422/1467 (28.77%)	123/365 (33.7%)	545/1832 (29.75%)
Severe malnutrition	139/1467 (9.48%)	38/365 (10.41%)	177/1832 (9.66%)
Sickle cell disease	41/1467 (2.79%)	12/365 (3.29%)	53/1832 (2.89%)
Asthma	160/1467 (10.91%)	35/365 (9.59%)	195/1832 (10.64%)
Cardiac disease	15/1467 (1.02%)	10/365 (2.74%)	25/1832 (1.36%)
Genitourinary disease	10/1467 (0.68%)	5/365 (1.37%)	15/1832 (0.82%)
Diabetes	3/1467 (0.2%)	1/365 (0.27%)	4/1832 (0.22%)
Neurological disorder	116/1467 (7.91%)	18/365 (4.93%)	134/1832 (7.31%)
Sepsis	8/1467 (0.55%)	1/365 (0.27%)	9/1832 (0.49%)
Diarrhoea and dehydration	79/1467 (5.39%)	25/365 (6.85%)	104/1832 (5.68%)
Acute neurological disorder	158/1467 (10.77%)	39/365 (10.68%)	197/1832 (10.75%)

*Denominators less than these values indicate missing data.

Using the predefined study criteria, we assigned a severity category to 1799 (98.2%) patients whose records had adequate documentation of clinical signs. On the basis of clinical characteristics, we identified 588 (32.7%) with severe pneumonia, younger children (aged 5–9 years) were more likely to fulfil this definition (p=0.012). Oxygen saturations were recorded for 812 (44.3%) children and there was significant interhospital variation (range 9.9%–82.3%, median, 43.8%). Where oxygen saturations were recorded, the median value was 95% (IQR 90%–98%) and 180 (22.2%) had saturations below 90%. Pallor was a common clinical finding—detected in 340/1760 (19.3%) children. Pallor was observed more frequently in older children (aged 10–14 years, p≤0.001) and in high malaria transmission regions (226/816 (27.7%) compared with 114/944 (12.1%) children in low-transmission regions (p≤0.001)). HIV status was determined to be positive in 9.9% of patients and was also found more frequently in older children (p≤0.001).

### Outcome analysis

Outcome information was available for 1825/1832 (99.5%) patients, of which 145 (7.9%) died. Inpatient case fatality was higher in children aged 10–14 years. Univariate analysis indicated that mortality increased proportionate to the degree of pallor (table 4) with severe pallor (compared with none) associated with an OR for death of 9.57 (95% CI 5.94 to 15.41) and a pneumonia case fatality of 30.1%. Other clinical characteristics strongly associated with death in unadjusted analyses (OR >2) were: reduced consciousness, mild/moderate pallor; the inability to eat or drink; central cyanosis; severe pneumonia; oxygen saturations <90%; acidotic breathing and grunting. For comorbidities: acute neurological disorder; sepsis; HIV and severe malnutrition were strongly associated with death (OR >2), while malaria and sickle cell disease were not. Wheeze and asthma were protective.

**Table 4:** Univariate associations for mortality among all children admitted to the study hospitals with pneumonia

Characteristic	Number of deaths/number of patients	Mortality	OR (95% CI)	P value
Age 10–14 years	51/363	14.05%	2.38 (1.66 to 3.42)	<0.001
Age 5–9 years	94/1462	6.43%		
Male	75/1019	7.36%	0.83 (0.59 to 1.16)	0.318
Female	70/800	8.75%		
High malaria transmission region	85/846	10.05%	1.71 (1.21 to 2.41)	0.003
Low malaria transmission region	60/979	6.13%		
Referred	48/322	14.91%	2.63 (1.78, 3.86)	<0.001
Not referred	74/1183	6.26%		
Readmitted	13/279	4.66%	0.55 (0.31 to 1.00)	0.063
Not readmitted	101/1243	8.13%		
Temperature ≥38.0°C	47/667	7.05%	0.94 (0.64 to 1.38)	0.83
Temperature <38.0°C	72/966	7.45%		
Respiratory rate >30	94/1126	8.35%	1.92 (1.13 to 3.27)	0.019
Respiratory rate ≤30	17/376	4.52%		
Central cyanosis	5/25	20%	3.20 (1.18 to 8.67)	0.033
No central cyanosis	126/1739	7.25%		
Grunting	35/270	12.96%	2.15 (1.42 to 3.24)	<0.001
No grunting	95/1465	6.48%		
Acidotic breathing	10/70	14.29%	2.21 (1.10 to 4.43)	0.04
No acidotic breathing	116/1655	7.01%		
Reduced consciousness	37/128	28.91%	6.65 (4.30 to 10.27)	<0.001
Alert	93/1613	5.77%		
Cannot eat or drink	47/296	15.88%	3.42 (2.31 to 5.07)	<0.001
Can eat and drink	72/1378	5.22%		
Stridor	7/58	12.07%	1.75 (0.78 to 3.93)	0.268
No stridor	119/1633	7.29%		
Difficulty breathing	82/900	9.11%	1.47 (1.03 to 2.10)	0.04
No difficulty breathing	55/862	6.38%		
Wheeze	8/223	3.59%	0.43 (0.20 to 0.88)	0.026
No wheeze	122/1516	8.05%		
Crackles	59/613	9.62%	1.59 (1.11 to 2.29)	0.014
No crackles	70/1118	6.26%		
Severe pallor	34/113	30.09%	9.57 (5.94 to 15.41)	<0.001
Mild/Moderate pallor	40/224	17.86%	4.83 (3.15 to 7.41)	<0.001
No pallor	61/1417	4.30%	1	
Chest wall indrawing	65/711	9.14%	1.45 (1.01 to 2.07)	0.05
No chest wall indrawing	67/1031	6.50%		
Convulsions	37/252	14.68%	2.48 (1.65 to 3.72)	<0.001
No convulsions	97/1495	6.49%		
Oxygen saturations <90%	25/179	13.97%	2.76 (1.61 to 4.76)	<0.001
Oxygen saturations ≥90%	35/631	5.55%		
Mid-upper arm circumference <115 mm	8/47	17.02%	3.81 (1.66 to 8.77)	0.002
Mid-upper arm circumference <115 mm	35/685	5.11%		
Severe pneumonia	77/583	13.21%	2.86 (2.01 to 4.07)	<0.001
Non-severe pneumonia	61/1209	5.05%		
HIV positive	33/181	18.23%	3.05 (2.00 to 4.66)	<0.001
Not HIV positive	112/1644	6.81%		
Malaria diagnosed	48/542	8.86%	1.19 (0.83 to 1.71)	0.401
No malaria diagnosed	97/1283	7.56%		
Severe malnutrition	30/177	16.95%	2.72 (1.76 to 4.21)	<0.001
No severe malnutrition	115/1648	6.98%		
Sickle cell disease	7/53	13.21%	1.80 (0.80 to 4.07)	0.238
No sickle cell disease	138/1772	7.79%		
Asthma	2/195	1.03%	0.11 (0.03 to 0.44)	<0.001
No asthma	143/1630	8.77%		
Cardiac disease	5/25	20%	2.96 (1.10 to 8.02)	0.043
No cardiac disease	140/1800	7.78%		
Renal disease	2/15	13.33%	1.79 (0.40 to 8.03)	0.337
No renal disease	143/1810	7.90%		
Diabetes	1/4	25%	3.88 (0.40 to 37.56)	0.282
No diabetes	144/1821	7.91%		
Chronic neurological disorder	7/134	5.22%	0.62 (0.28 to 1.35)	0.296
No chronic neurological disorder	138/1691	8.16%		
Sepsis	3/9	33.33%	5.89 (1.46 to 23.82)	0.029
No sepsis	142/1816	7.82%		
Diarrhoea with dehydration	14/104	13.46%	1.89 (1.05 to 3.41)	0.051
No diarrhoea with dehydration	131/1721	7.61%		
Acute neurological disorder	53/194	27.32%	6.29 (4.30 to 9.19)	<0.001
No acute neurological disorder	92/1631	5.64%		

In multivariate analyses (table 5), we adjusted for hospital, sex, age, respiratory rate >30/min and the presence of: central cyanosis, grunting, reduced consciousness, inability to eat or drink, wheeze, crackles, pallor and indrawing (*model 1*). Difficulty breathing, acidotic breathing and convulsions were excluded due to collinearity with other variables. We excluded temperature and stridor, as neither had associations with death in univariate analysis. We were unable to include the data on oxygen saturation in our models, even after multiple imputation because the measurement of pulse oximetry was only recorded for 44.3% of children and varied substantially over time and between hospitals. This was due to inconsistency in availability of pulse oximeters as a result of factors such as damage, failure, loss or interrupted use in cases where they belonged to individual clinicians. Additional information pertaining to HIV status, malaria diagnosis and the presence of severe malnutrition were added in *model 2*. There was consistency in the point estimates for the associations analysed in the multiple imputation models and the complete case analysis (online supplementary table 2), so we have reported the findings from the multiple imputation models to maximise use of the available data. The presence of severe pallor, reduced consciousness, mild/moderate pallor, central cyanosis and older age (>9 years) were strongly associated with death (adjusted OR (aOR) >2). Respiratory rate >30/min and inability to eat or drink were also significantly associated with poor outcome. In *model 2*, HIV (aOR 2.31, 95% CI 1.39 to 3.84) and severe malnutrition (aOR 1.89, 95% CI 1.12 to 3.21) (*model 2*) were associated with mortality and malaria was not. In this model, central cyanosis and respiratory rate >30 lost significance in their association with death. In both models, sex and the presence of grunting, crackles and indrawing were not associated with mortality and wheeze was found to be protective, although not significantly.

**Table 5:** Multivariate models for risk factors for mortality for all pneumonia cases (with multiple imputation)

Variable	Model 1			Model 2		
	OR	95% CI	P value	OR	95% CI	P value
Sex: male	0.87	0.6 to 1.27	*0*.*477*	0.84	0.57 to 1.23	*0*.*362*
Age: >9	2.78	1.83 to 4.24	*<* *0*.*001*	2.48	1.62 to 3.80	*<* *0*.*001*
Respiratory rate: >30	1.87	1.02 to 3.43	*0*.*044*	1.67	0.94 to 2.98	*0*.*083*
Cyanosis: true	3.27	1.03 to 10.43	*0*.*045*	2.89	0.86 to 9.75	*0*.*086*
Grunting: true	1.48	0.89 to 2.49	*0*.*134*	1.69	0.99 to 2.88	*0*.*054*
Reduced consciousness: true	3.65	2.07 to 6.44	*<* *0*.*001*	4.18	2.34 to 7.47	*<* *0*.*001*
Cannot eat or drink: true	1.82	1.13 to 2.93	*0*.*013*	1.91	1.17 to 3.13	*0*.*01*
Wheeze: true	0.52	0.23 to 1.16	*0*.*107*	0.55	0.24 to 1.3	*0*.*172*
Crackles: true	1.42	0.93 to 2.17	*0*.*101*	1.3	0.83 to 2.03	*0*.*255*
Pallor: severe	8.06	4.72 to 13.75	*<* *0*.*001*	7.4	4.28 to 12.8	*<* *0*.*001*
Pallor: mild/moderate	3.5	2.19 to 5.6	*<* *0*.*001*	2.96	1.83 to 4.79	*<* *0*.*001*
Indrawing: true	1.34	0.85 to 2.1	*0*.*211*	1.2	0.77 to 1.89	*0*.*42*
HIV: positive	–	–	–	2.31	1.39 to 3.84	*0*.*001*
Malaria: positive	–	–	–	0.81	0.49 to 1.32	*0*.*386*
Severe malnutrition: present	–	–	–	1.89	1.12 to 3.21	*0*.*018*

Pallor (any degree) performed better than all other individual clinical signs (AUC 0.69, 95% CI 0.64 to 0.74) in predicting mortality, and generally, the presence of individual clinical signs was a poor predictor of death (AUC range 0.51–0.69) (online supplementary table 1). Simple combinations of pallor with other clinical signs that had strong correlation with poor outcome improved the predictive performance: for example, pallor and/or reduced consciousness (AUC 0.73, 95% CI 0.69 to 0.78) and pallor and/or reduced consciousness and/or central cyanosis (AUC 0.74, 95% CI 0.69 to 0.79).

### Subgroup analyses

In *model 3* (online supplementary material), we examined the subset of children aged 5–10 years (n=1585) for whom we were able to calculate WAZ scores (n=1496) and found that a score of ≤−3 SD was associated with mortality (aOR 2.99, 95% CI 1.61 to 5.55). Other factors associated with death were consistent with the findings shown in *models 1* and *2*. In a sensitivity analysis of children admitted to hospitals in regions of low malaria endemicity, where malaria is unlikely to account for anaemia, we found that severe pallor and mild/moderate pallor were still significantly associated with mortality (aOR 12.12, 95% CI 4.12 to 35.67 and aOR 2.65, 95% CI 1.09 to 6.43, respectively) (*model 4*, online supplementary material).

### Antibiotic treatment

**Figure 3:**
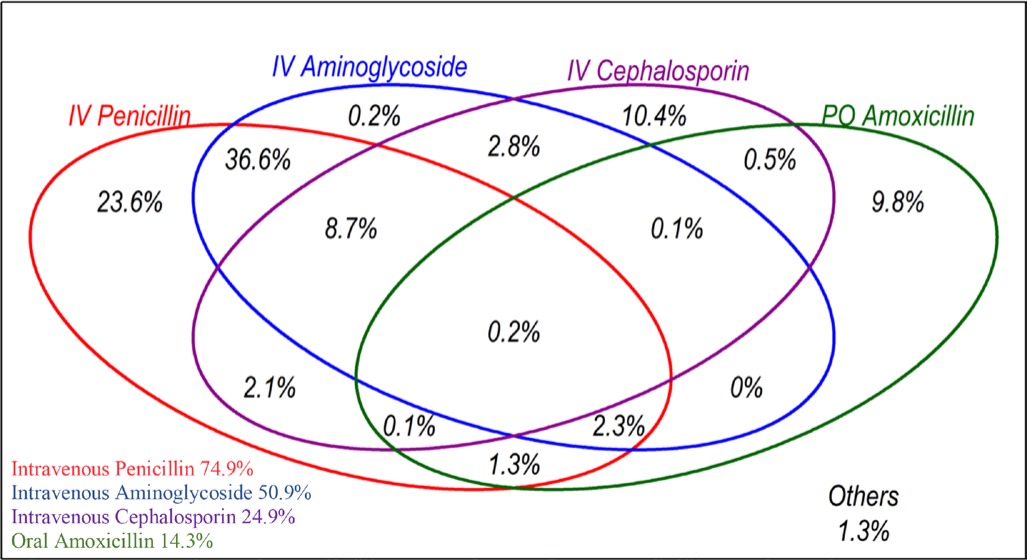
Proportion of children with pneumonia in the study hospitals who were prescribed at least one of the top four most frequently prescribed antibiotics on admission to hospital (n=1725).

There were 3172 documented antibiotic prescriptions for 1725 children on admission, with 22 different antibiotics used (online supplementary table 3). The most frequently prescribed antimicrobials were intravenous penicillin (1291 prescriptions), intravenous aminoglycosides (879), intravenous cephalosporins (430) and oral amoxicillin (247) often in combinations. Over 98% of children were prescribed at least one of these four drugs (figure 3) and 85% were prescribed at least one of the WHO first-line drugs (table 1). Excluding children with HIV or severe malnutrition and those who were re-admitted or referred (who may not typically get first-line antibiotic regimens) there were 1057 children, of whom 523 (49.5%) were treated in keeping with WHO under 5 guidance: penicillin and gentamicin for severe pneumonia; amoxicillin or penicillin for non-severe pneumonia.

## Discussion

### Mortality

Children aged 5–14 years with pneumonia accounted for 11.7% of all medical admissions to the study hospitals for children aged >5 and<15 years. Overall, we found that mortality was high (7.9%) in this group. There were more admissions and deaths in children aged 5–9 years, but there was higher case fatality among those aged 10–14 years (14.1%). Pneumonia case fatality rates (CFRs) have not been reported before in these age groups and, perhaps surprisingly they are similar to those reported in hospital-based pneumonia studies of children under 5 years: studies from sub-Saharan Africa report rates ranging from 5% to 10.4%.^18 21–25^ These include pneumonia mortality rates from studies within the CIN: for children aged between 2 and 59 months one study reported CFR of 5.9%,^21^ and another reported 5%, the latter excluded children with severe acute malnutrition, meningitis and HIV.^18^

These higher than expected CFRs could be explained by several factors: (1) late presentation to hospital,^26 27^ (2) different admission thresholds or inadequate guidance for primary care workers on what constitutes severe illness in this age group. This may contribute to late presentation or could result in only the highest-risk children being admitted—there were low absolute number of children admitted (especially in those aged 10–14 years) if we consider that the general population cohort is the same size as for under 5s, (3) residual pneumococcal disease—pneumococcal vaccination was only introduced in Kenya in 2011, therefore the majority of children in our study would not have been immunised making them more susceptible to invasive disease,^28^ although they are likely to have benefited from herd immunity,^29^ (4) a wider range of pneumonia pathogens that are not susceptible to commonly used antibiotics and (5) a high proportion of children with comorbidities such as HIV or underlying chronic illness (including some that may not be detected due to weak diagnostic capacity).

### Clinical risk factors

The characteristics associated with mortality in our study: pallor (any), central cyanosis, reduced consciousness, tachypnoea, inability to eat or drink, HIV and severe malnutrition are broadly similar to those described in studies for children under 5 years. Pallor stood out from other individual characteristics in the strength of its association with death. It is a widely accepted clinical tool used to identify children with moderate and severe anaemia in resource-limited settings (although the majority of the data supporting its use are derived from studies in under 5s).^30–33^ This association with mortality has previously been shown for children under 5 years but never before in older children.^18 22 34 35^ Children in our cohort may be anaemic for a number of reasons: haemolysis due to malaria, ^30^ atypical infection or sepsis; myelosuppression related to malaria and co-infection with non-typhoidal salmonellae^36^; nutritional deficiency or other comorbidities such as HIV, sickle cell and malignancy. However, the relationship between anaemia, pneumonia and high mortality is less clear. Does the presence of anaemia simply represent children with comorbidities or those living in extreme poverty or neglect—children for whom we might expect worse health outcomes? In our analysis, socioeconomic factors were not accounted for. Children may also have been referred to these higher-level facilities specifically for blood transfusion, therefore anaemic children may be over-represented in our study cohort. However, the fact that even mild forms of clinical pallor remained associated with death counters this. Alternatively, there may be a specific physiological relationship between anaemia and respiratory compromise that affects clinical outcome. For example, in the context of pneumonia, anaemia might compound respiratory compromise further by reducing the oxygen carrying capacity of red blood cells.^37^ Finally, severe anaemia itself is a well-recognised cause of respiratory distress, therefore it is possible that children in our study were misdiagnosed with pneumonia. Again, the association of mild/moderate anaemia with death counters this. It is likely that the role of anaemia is complex and multifactorial and this relationship warrants further study.

Our initial analysis shows that both individual and simple combinations of clinical signs do not have good sensitivity or specificity in predicting clinical outcome among hospitalised over 5s with pneumonia (online supplementary table 1). For example, the combination of clinical characteristics used to define severe pneumonia by WHO in the under 5s was poor in discriminating those at risk of death (sensitivity: 0.56, specificity: 0.68 and AUC: 0.62). Any pallor (AUC 0.69), or combinations of pallor and/or reduced consciousness (AUC 0.73) or pallor and/or reduced consciousness and/or central cyanosis (AUC 0.74) performed better than having ‘severe pneumonia’. However, in our data mortality was high in children without signs of severe disease (5.1%), severe pneumonia was observed more frequently in children aged 5–9 years—the group with lower mortality—and we have no data on the clinical signs among similarly aged outpatient children presenting with possible pneumonia. Further work is therefore needed to explore and then test which clinical signs may be useful in identifying high-risk children among those presenting with possible pneumonia at ages 5–14 years.

### Human immunodeficiency virus

HIV tests were ordered in 1027 (56.1%) children. The proportion with a confirmed diagnosis was high, particularly in those >9 where 16.4% were HIV positive. This is much higher than population prevalence rates reported in Kenya, which are estimated at 0.9% for children aged 18 months to 14 years.^38^ The high rates we observed could be because pneumonia disproportionately affects patients with HIV.^39 40^ However, it is also plausible that population prevalence of HIV is higher in this older population—they are less likely to have benefited from prevention of mother-to-child transmission programmes (first officially launched in Kenya in 2002), and may have some risk of HIV acquisition through sexual transmission. In our cohort HIV was associated with death, which is consistent with other studies.^41^ Factors that might contribute to poor outcome in these HIV-infected children include complex sociocultural issues around stigma and disclosure and access to and retention in care. Unfortunately, our routine dataset does not allow us to determine whether these children are enrolled in HIV care and treatment programmes nor provide information on markers of immune function such as CD4 count and viral load.

### Treatment

In our study, the majority (85%) of children who were prescribed an antibiotic on admission were prescribed at least one of the first-line antibiotics recommended by the current WHO pneumonia case management guidelines. This may indicate that clinicians treat children over 5 years as they do those under 5 years (who represent the majority of pneumonia cases that they see), or that clinicians do reference the under 5 guidelines when treating older children. These prescribing practices are also likely to be reinforced by factors such as the low cost and availability of these drugs, clinician experience and familiarity using them,^42^ and regular audit and feedback against national protocols (for under 5s).^12^ However, these treatments do not adequately target organisms which can cause complicated pneumonia such as *Staphylococcus aureus* or atypical pathogens such as *Mycoplasma pneumoniae*. There is some evidence to suggest that over 5s are more likely to present with atypical infections, these studies are from high-income settings with small sample sizes.^7^

The lack of robust evidence on pneumonia aetiology in this age group means that best practice with regard to antibiotic treatment really is unknown. The recently completed Pneumonia Aetiology Research for Child Health study sought to refresh understanding of paediatric pneumonia aetiology in LMICs, but focused exclusively on under 5s.^43^ Unfortunately, we were unable to confirm aetiology in our study due to the limited diagnostic capacity of the study hospitals.^8^ There is an urgent need for carefully designed studies on the effectiveness of alternative antibiotics, as well as other supportive care regimens such as oxygen and blood transfusion,^44 45^ to guide the development of empirical treatment protocols for pneumonia in older children.

### Strengths and limitations

We collected data from routine clinical practice, representing real-life scenarios rather than a controlled research environment, and our large sample size was taken from multiple hospitals across Kenya. As a result, our data are the most representative to date of children aged 5–14 years admitted to district hospitals in sub-Saharan Africa. To our knowledge, this is the first study that examines specifically for risk factors in over 5s in a resource-poor setting. We examined a complete 4-year period to reduce seasonal bias—an important consideration in the context of respiratory infections.^46^

Our analysis is based on the documentation of clinical findings with retrospective data entry. This exposes the study to potential bias due to missing data—either due to gaps in medical documentation or misplaced hospital records. Considerable effort was taken to mitigate this and ensure comprehensive sampling of high-quality data.^9^ This included immediate data entry at the point of discharge, regular audit of clinical records against hospital admission registers, training and supervision of data clerks, standardised patient admission forms for clinicians with regular feedback on their completeness and dissemination of standard clinical guidelines to healthcare workers within the study hospitals. We also applied multiple imputation to address missing data and maximise the use of available data. Our CIN provides a suitable platform for a future prospective study examining the findings highlighted in this analysis while minimising the limitations described above.

The observational nature of this study and use of data collected during routine care also presents limitations: the interpretation of clinical signs is likely to vary because characteristics are identified and recorded by a variety of junior clinicians.^47^ Although, arguably, this may also increase the generalisability of our findings. There is potential for misdiagnosis and misclassification because of limited access to diagnostic equipment. For example, the lack of pulse oximetry data in the study may have led to an underestimation of the utility of WHO severity classification. We were unable to account for the effect of antibiotic treatment on outcome because we only had information on *prescriptions* on *admission* which does not reflect what was actually administered, nor any changes to treatment made during admission. Similarly, we were unable to reliably assess the impact of quality of care and other unobserved factors that might influence management decisions, such as the clinician’s gut feeling influencing admission decisions.^48^

Finally, we did not have data on children managed as outpatients or in the community. This group is likely to represent the majority of over 5s with pneumonia and being able to compare the two groups would further elucidate risk factors for poor outcome. Future work should also focus on broader risk factors for death that have been highlighted in previous studies (for children under 5 years), these include low maternal education and other socioeconomic factors such as indoor pollution.^41^

## Conclusion

Children over 5 years are a neglected group with high inpatient pneumonia mortality. We have shown non-trivial risk in this population and that there is a need for research to explore the underlying factors that account for this, particularly in older children (aged 10–14 years). Apparently important signs such as pallor or reduced consciousness are not included in the WHO criteria for classification of severity for under 5s and so directly extending this case management approach to over 5s does not seem appropriate. Greater attention may also need to be paid to comorbidity in these age groups, especially with HIV and severe malnutrition. Our data do not constitute formal evidence that using these patient features to indicate high risk of mortality, and thus alternative or aggressive treatments, would save lives. Rather our data make it clear that we need to better understand the characteristics of children presenting with possible pneumonia at all levels of the health system ideally linked to studies of aetiology. Furthermore, in the 5–14 years age group there is need to explore the aetiology and pathophysiology of anaemia among children with respiratory distress; care-seeking behaviour and adequacy of health services delivery, broader socioeconomic risk factors as well as exploring which interventions are most effective at improving outcomes. This agenda is important as we seek to implement universal health coverage that should span children of all ages.
