# Supplementary material for: Risk factors for death among children aged 5–14 years hospitalised with pneumonia: a retrospective cohort study in Kenya
Source: BMJ Glob Health. 2019 Sep 3;4(5):e001715. doi: 10.1136/bmjgh-2019-001715 (PMC6730574; doi:10.1136/bmjgh-2019-001715)
Supplement: Supplementary data [file bmjgh-2019-001715supp001.pdf]

Supplementary material

Table 1: Sensitivity, specificity, positive likelihood ratio (LR+) and negative likelihood ratio (LR-) of clinical characteristics in predicting death among children presenting to the study hospitals with pneumonia

Clinical Characteristics	AUC (95% CI)	Sensitivity	Specificity	LR+	LR-
**Sex: male**	0.50 (0.42 - 0.58)	0.52	0.44	0.92	1.11
**Age: >9**	0.58 (0.53 - 0.63)	0.35	0.81	1.89	0.80
**RR: >30**	0.55 (0.49 - 0.61)	0.85	0.26	1.14	0.59
**RR: >40**	0.57 (0.52 - 0.63)	0.62	0.53	1.32	0.71
**RR: >50**	0.58 (0.52 - 0.64)	0.41	0.76	1.70	0.78
**Central cyanosis: present**	0.51 (0.47 - 0.54)	0.04	0.99	3.12	0.97
**Grunting: present**	0.56 (0.52 - 0.61)	0.27	0.85	1.84	0.86
**Reduced consciousness: present**	0.62 (0.55 - 0.68)	0.28	0.94	5.04	0.76
**Inability to eat and drink: present**	0.62 (0.55 - 0.68)	0.39	0.84	2.47	0.72
**Wheeze: absent**	0.53 (0.51 - 0.56)	0.94	0.13	1.08	0.46
**Crackles: present**	0.56 (0.49 - 0.62)	0.46	0.65	1.32	0.83
**Any pallor: present**	0.69 (0.64 - 0.74)	0.55	0.84	3.37	0.54
**Severe pallor: present**	0.60 (0.56 - 0.64)	0.25	0.95	5.16	0.79
**Indrawing: present**	0.54 (0.50 - 0.59)	0.49	0.60	1.23	0.85
**HIV: positive**	0.57 (0.53 - 0.61)	0.23	0.91	2.58	0.85
**Malaria: positive**	0.50 (0.45 - 0.56)	0.33	0.71	1.13	0.95
**Severe malnutrition: present**	0.56 (0.52 - 0.60)	0.21	0.91	2.36	0.87
**Severe pneumonia: present**	0.62 (0.57 - 0.68)	0.56	0.68	1.75	0.65
**Any one of: Any pallor: present, reduced consciousness: present**	0.73 (0.69 - 0.78)	0.68	0.79	3.29	0.40
**Any one of: Any pallor: present, reduced consciousness: present, central cyanosis: present**	0.74 (0.69 - 0.79)	0.71	0.78	3.24	0.37
**Any one of: Any pallor: present, reduced consciousness: present, central cyanosis: present, age: >9**	0.72 (0.68 - 0.77)	0.81	0.63	2.21	0.29

Macpherson L, *et al. BMJ Global Health* 2019; 4:e001715. doi: 10.1136/bmjgh-2019-001715

Table 2: Multivariate models for risk factors for mortality for all pneumonia cases (complete case analysis)

	Model 1			Model 2		
**Variable**	**Odds ratio**	**95% Confidence interval**	***p value***	**Odds ratio**	**95% Confidence interval**	***p value***
**Sex: male**	1.04	0.64 - 1.69	*0.872*	1	0.61 - 1.64	*0.988*
**Age: >9**	2.7	1.58 - 4.61	*<0.001*	2.32	1.34 - 4.04	*0.003*
**RR: >30**	2.29	1.16 - 4.5	*0.016*	2.26	1.12 - 4.55	*0.023*
**Cyanosis: true**	4.81	1.18 - 19.7	*0.029*	5.18	1.15 - 23.35	*0.032*
**Grunting: true**	0.95	0.5 - 1.81	*0.873*	1.12	0.57 - 2.18	*0.742*
**Reduced consciousness: true**	4.61	2.35 - 9.05	*<0.001*	5.78	2.85 - 11.72	*<0.001*
**Cannot drink: true**	1.92	1.08 - 3.42	*0.027*	1.96	1.08 - 3.54	*0.026*
**Wheeze: true**	0.18	0.04 - 0.75	*0.019*	0.19	0.04 - 0.83	*0.028*
**Crackles: true**	1.57	0.94 - 2.62	*0.082*	1.37	0.81 - 2.31	*0.241*
**Pallor: severe**	9	4.7 - 17.25	*<0.001*	7.61	3.83 - 15.09	*<0.001*
**Pallor: mild/mod**	3.24	1.81 - 5.78	*<0.001*	2.55	1.39 - 4.69	*0.003*
**Indrawing: true**	1.41	0.83 - 2.4	*0.2*	1.22	0.70 - 2.11	*0.482*
**HIV +**	-	-	-	2.76	1.49 - 5.09	*0.001*
**Malaria +**	-	-	-	0.81	0.43 - 1.52	*0.507*
**Severe malnutrition: present**	-	-	-	2.63	1.41 - 4.93	*0.003*

Macpherson L, *et al. BMJ Global Health* 2019; 4:e001715. doi: 10.1136/bmjgh-2019-001715

Model 3: Subgroup multivariate model for risk factors for mortality for children age 5-10 years (n= 1585) using weight for age Z (WAZ) score in place of severe malnutrition

	Model 3		
**Variable**	**Odds ratio**	**95% Confidence interval**	***p value***
**Sex: male**	0.86	0.48 – 1.54	*0.615*
**Age: >9**	-	-	-
**RR: >30**	1.89	0.85 – 4.23	*0.12*
**Cyanosis: true**	6.35	1.33 – 30.25	*0.02*
**Grunting: true**	0.98	0.46 – 2.07	*0.96*
**Reduced consciousness: true**	6.27	2.8 – 14.08	*<0.001*
**Cannot drink: true**	1.39	0.68 – 2.84	*0.367*
**Wheeze: true**	0.13	0.02 – 1.02	*0.053*
**Crackles: true**	1.19	0.65 – 2.18	*0.582*
**Pallor: severe**	9.89	4.68 – 20.93	*<0.001*
**Pallor: mild/mod**	2.85	1.35 – 6	*0.006*
**Indrawing: true**	1.21	0.65 – 2.27	*0.551*
**HIV +**	2.49	1.18 – 5.28	0.017
**Malaria +**	0.9	0.44 – 1.82	0.769
**WAZ ≤-3SD**	2.99	1.61 – 5.55	<0.001

Model 4: Subgroup multivariate model for risk factors for mortality for children presenting to study hospitals in regions of low malaria transmission

	Model 4		
**Variable**	**Odds ratio**	**95% Confidence interval**	***p value***
**Sex: male**	1.08	0.54 – 2.16	*0.828*
**Age: >9**	3.84	1.78 – 8.26	*<0.001*
**RR: >30**	2.53	0.86 – 7.45	*0.093*
**Cyanosis: true**	5.22	0.87 – 31.5	*0.072*
**Grunting: true**	1.26	0.51 – 3.12	*0.612*
**Reduced consciousness**	3.74	1.26 – 11.05	*0.017*
**Cannot drink: true**	1.98	0.92 – 4.24	*0.079*
**Wheeze: true**	0.17	0.22 – 1.3	*0.088*
**Crackles: true**	1.12	0.53 – 2.37	*0.77*
**Pallor: severe**	12.12	4.12 – 35.67	*<0.001*
**Pallor: mild/mod**	2.65	1.09 – 6.43	*0.031*
**Indrawing: true**	1.33	0.62 – 2.87	*0.47*

Macpherson L, *et al. BMJ Global Health* 2019; 4:e001715. doi: 10.1136/bmjgh-2019-001715

Table 3: Admission antibiotic prescriptions for all children admitted to the study hospitals

Antibiotic	Antibiotic class	Number of prescriptions
Penicillin (IV)	Beta-lactamase sensitive penicillins	1291
Penicillin V (PO)	Beta-lactamase sensitive penicillins	4
Gentamicin	Aminoglycoside	848
Amikacin	Aminoglycoside	58
Ceftriaxone	Third-generation cephalosporin	423
Ceftazidime	Third-generation cephalosporin	7
Cefuroxime	Second-generation cephalosporin	1
Amoxicillin	Penicillins with extended spectrum	247
Co-trimoxazole	Combinations of sulphonamides and trimethoprim	116
Metronidazole	Imidazole derivatives	74
Erythromycin	Macrolides	34
Azithromycin	Macrolides	10
Clarithromycin	Macrolides	3
Flucloxacillin	Beta-lactamase resistant penicillins	22
Co-amoxiclav	Combination of penicillins	13
Ciprofloxacin	Fluoroquinolones	9
Tetracycline	Tetracyclines	4
Vancomycin	Glycopeptides	3
Meropenem	Carbapenems	2
Chloramphenicol	Amphenicols	1
Clindamycin	Lincosamides	1
Nitrofurantoin	Nitrofuran derivatives	1

Macpherson L, *et al. BMJ Global Health* 2019; 4:e001715. doi: 10.1136/bmjgh-2019-001715
